# Supplementary figures and images for: SARS-CoV-2 ORF6 disrupts innate immune signalling by inhibiting cellular mRNA export
Source: PLoS Pathog. 2022 Aug 25;18(8):e1010349. doi: 10.1371/journal.ppat.1010349 (PMC9451085; doi:10.1371/journal.ppat.1010349)

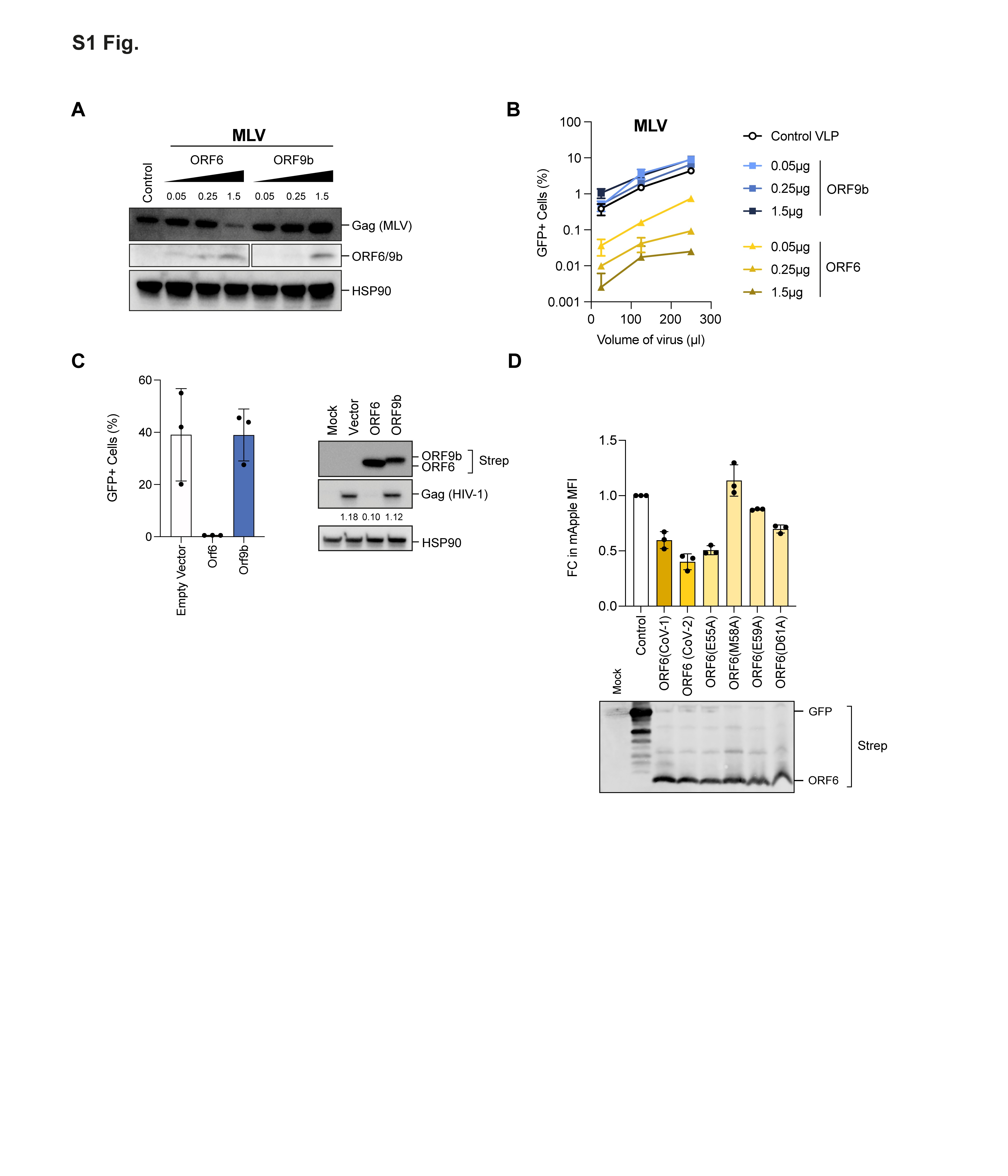

Supplement: S1 Fig — (A, B) To generate MLV VLPs, HEK293T cells were transfected with plasmids encoding Gag-Pol(MLV), VSV-G and either increasing amounts of pLVX-StrepII-ORF6 or pLVX-StrepII-ORF9b as well as a GFP reporter plasmid, pczCFG2fEGFPf. As a control, VLPs were generated with an empty pLVX vector. (A) Transfected cells were lysed and analysed by SDS-PAGE and immunoblotting for Gag(MLV), ORF6 or ORF9b and HSP90. (B) MDTF cells were infected with increasing amounts of MLV VLPs. Three days post infection, the percentage of GFP positive cells was determined by flow cytometry. Graph shows the mean and range of two biological repeats. (C) To generate HIV-1 VLPs, HEK293T cells were transfected with plasmids encoding Gag-Pol(HIV-1), VSV-G, a GFP reporter plasmid, pCSGW and pLVX-EF1α encoding either twin-strep tagged ORF6, ORF9b or as a control an empty retroviral plasmid. HeLa cells were infected with HIV-1 VLPs. Three days post infection, the percentage of GFP positive cells was determined by flow cytometry. From one biological repeat, cells were lysed and immunoblotted for anti-strep, Gag(HIV-1) and HSP90. Gag expression was quantified, normalised to HSP90, and shown in numbers below the Gag panel. (D) HEK293T cells were transfected with the pLVX-EF1α plasmid encoding either ORF6(CoV-1), ORF6(CoV-2) or the indicated ORF6 mutant as well as a plasmid encoding mApple. After 48h, cells were fixed and the mApple MFI measured by flow cytometry. The fold change (FC) in MFI is plotted relative to the control for independent biological repeats. Error bars show SEM. Transfected cell lysates were analysed by SDS-PAGE and immunoblotted with anti-strep. (TIF) [file ppat.1010349.s001.tif]

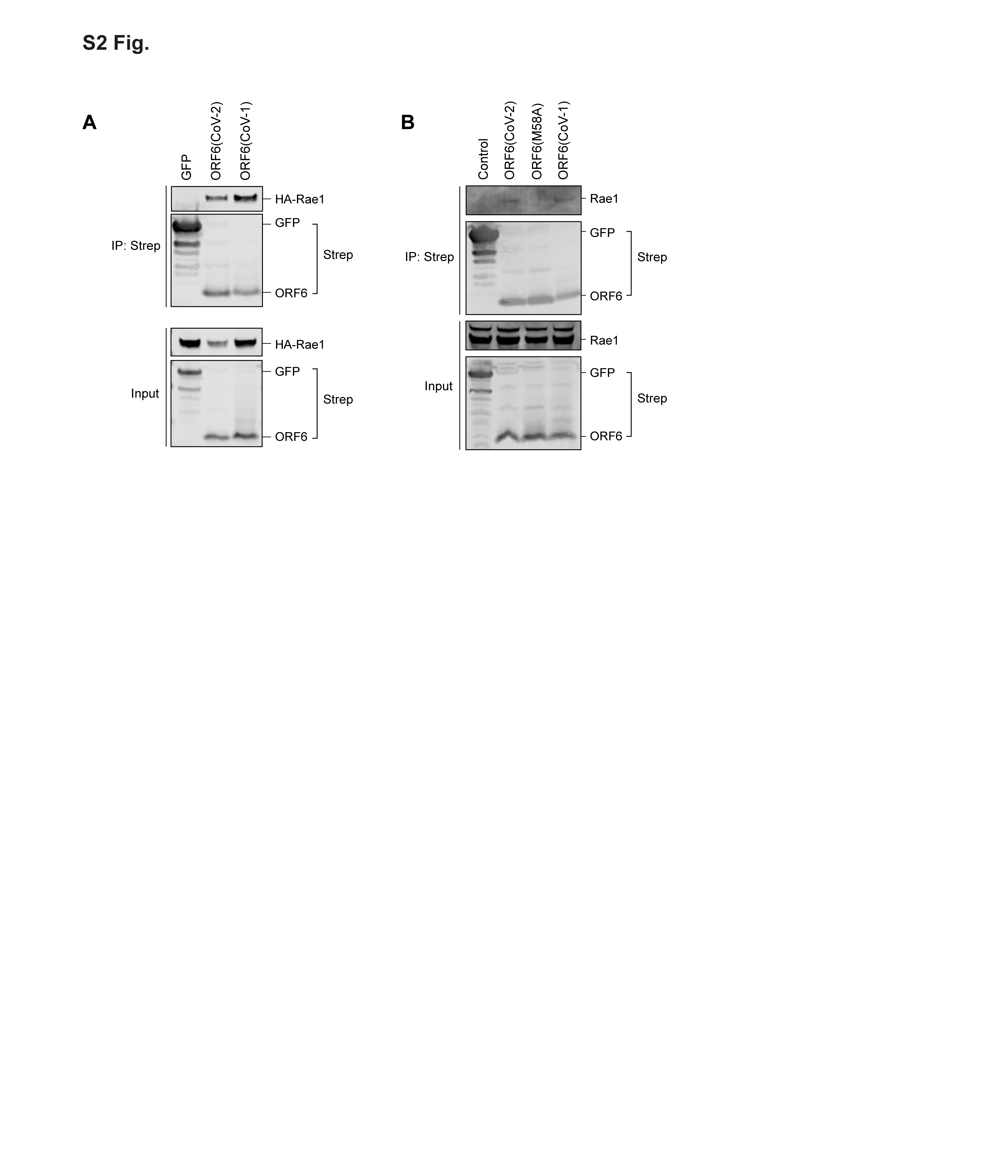

Supplement: S2 Fig — (A) HEK293T cells were transfected with plasmids encoding Twin-Strep-tagged GFP, ORF6(CoV-2) or ORF6(CoV-1) and HA-Rae1. After 24h, tagged proteins were immunoprecipitated with MagStrep beads and proteins eluted with biotin. Input lysates and eluate were separated by SDS-PAGE and analysed by immunoblotting for Strep, Nup98 and HA. (B) HEK293T cells were transfected with the pLVX-EF1α plasmid encoding Twin-Strep-tagged GFP or ORF6. After 24h, tagged proteins were immunoprecipitated with MagStrep beads and proteins eluted with biotin. Input lysates and eluate were separated by SDS-PAGE and immunoblotted for endogenous Rae1 and anti-Strep. (TIF) [file ppat.1010349.s002.tif]

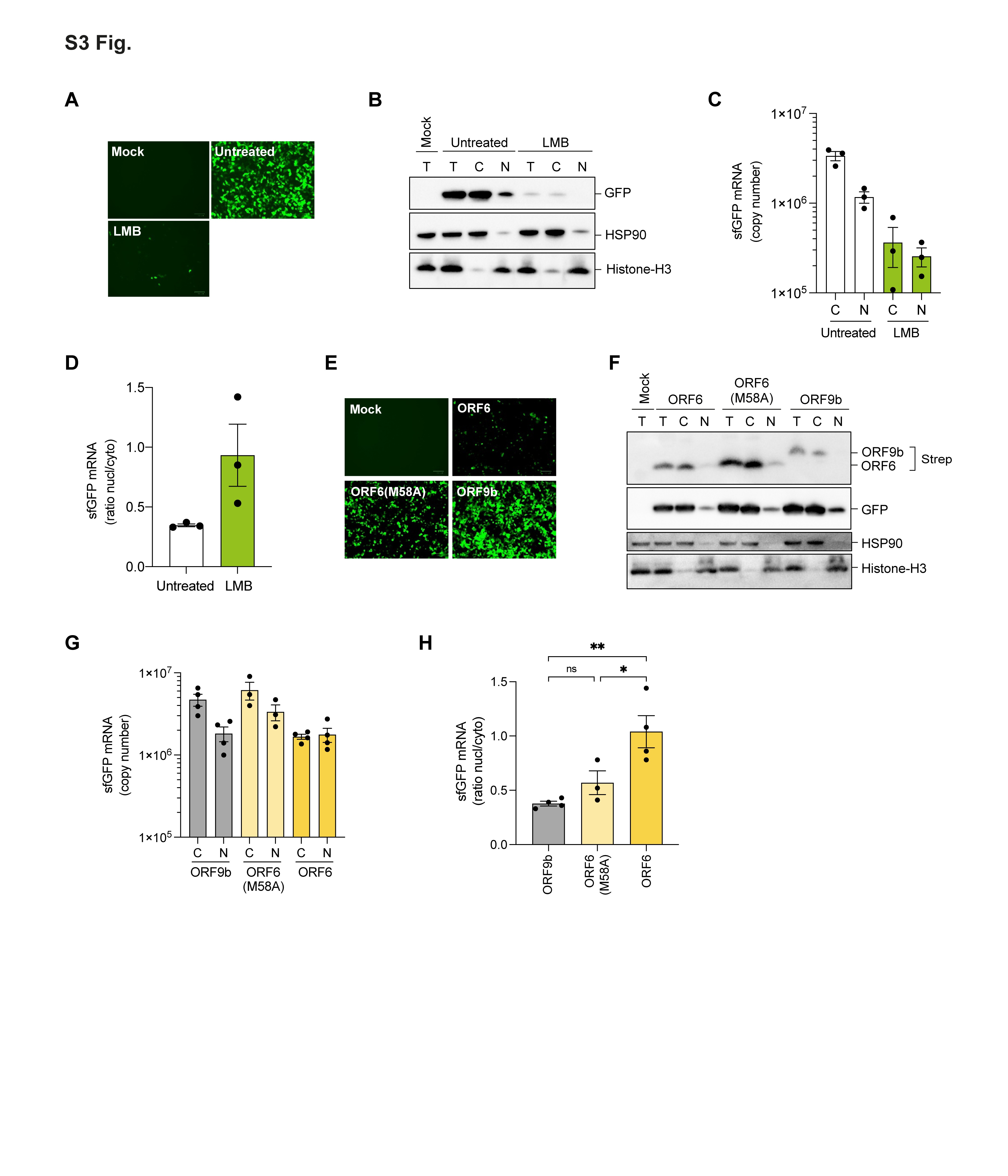

Supplement: S3 Fig — (A-D) HEK293T cells were transfected with a plasmid expressing GFP or left untransfected (mock) and either treated with 5 nM Leptomycin B (LMB) or left untreated. After 16h, cells were either harvested as a total cell lysate (T) or fractionated into Nucl (N) and Cyto (C) fractions. Total cell lysates and cellular fractions were divided into two for either protein analysis (B) or RNA extraction (C,D). (A) Low magnification fluorescent images showing GFP expression in transfected cells before harvest. (B) Protein levels were quantified by BCA assay, proportional amounts of the fractions related to the total cell lysate were analysed by immunoblotting for GFP, HSP90 (cytoplasmic marker) and Histone H3 (nuclear marker). (C) RNA from Nucl and Cyto fractions was converted into cDNA and GFP mRNA copy numbers measured by qPCR. (D) The ratio of Nucl to Cyto GFP mRNA was calculated and plotted for individual biological repeats. Error bars show the mean ± SEM. (E-H) HEK293T cells were co-transfected with plasmids encoding either ORF6, ORF6(M58A) or ORF9b and GFP, or left untransfected (mock). After 32h, cells were either harvested as a total cell lysate (T) or fractionated into Nucl (N) and Cyto (C) fractions. Total cell lysates and cellular fractions were divided into two for either protein analysis (F) or RNA extraction (G,H). (E) Low magnification fluorescent images showing GFP expression in transfected cells before harvest. (F) Protein levels were quantified by BCA assay, proportional amounts of the fractions related to the total cell lysate were analysed by immunoblotting for Strep, GFP, HSP90 (cytoplasmic marker) and Histone H3 (nuclear marker). (G) RNA from Nucl and Cyto fractions was converted into cDNA and GFP mRNA copy numbers measured by qPCR. (H) The ratio of Nucl to Cyto GFP mRNA was calculated and plotted for individual biological repeats. Error bars show the mean ± SEM. Significance was calculated by one-way ANOVA with Turkey’s post hoc test, *p<0.05, **p<0.01, [file ppat.1010349.s003.tif]

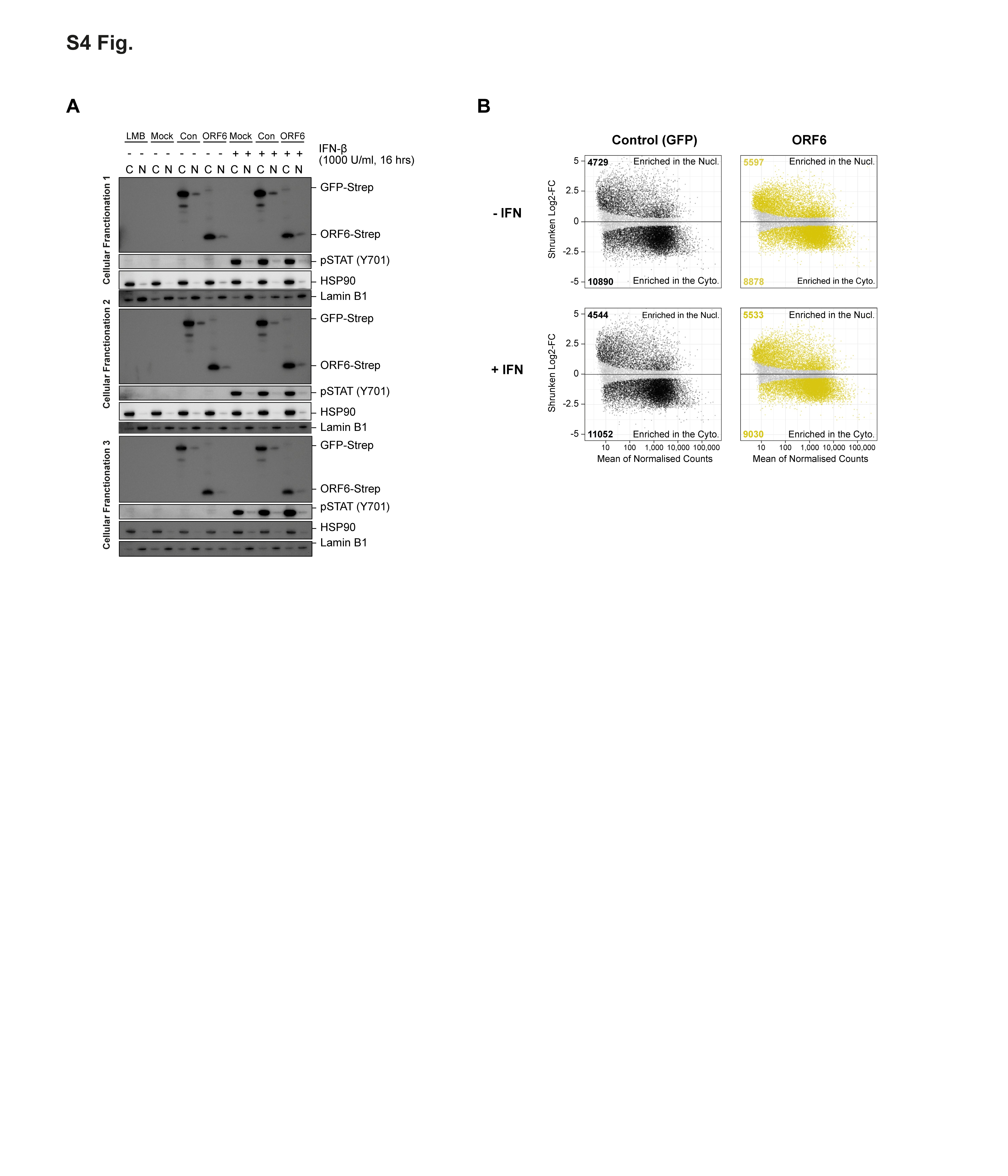

Supplement: S4 Fig — (A) The cell fractionations used in Fig 3A, and B were analysed by immunoblotting with anti-Strep to label ORF6 and GFP, anti-pSTAT(Y701) to confirm activation of the type-I IFN signalling cascade and anti-HSP90 (cytoplasmic marker) or anti-Lamin B1 (nuclear marker) to confirm successful fractionation. The analysis of three biological repeats is shown. (B) From the mRNAseq data in Fig 3. The log2-fold change (Log2-FC) in mRNA abundance was compared between the Nucl and Cyto fractions for both GFP and ORF6 expressing cells, without (upper panels) and with IFN treatment (lower panels). The log-2FC was weighted against the adjusted p-value (shrunken Log2-FC) to show mRNAs that are significantly enriched (GFP; black, ORF6; yellow) in either the cytoplasm or nucleus. The number of mRNAs significantly enriched are shown. The mRNA count was normalised and averaged between three biological repeats. (TIF) [file ppat.1010349.s004.tif]

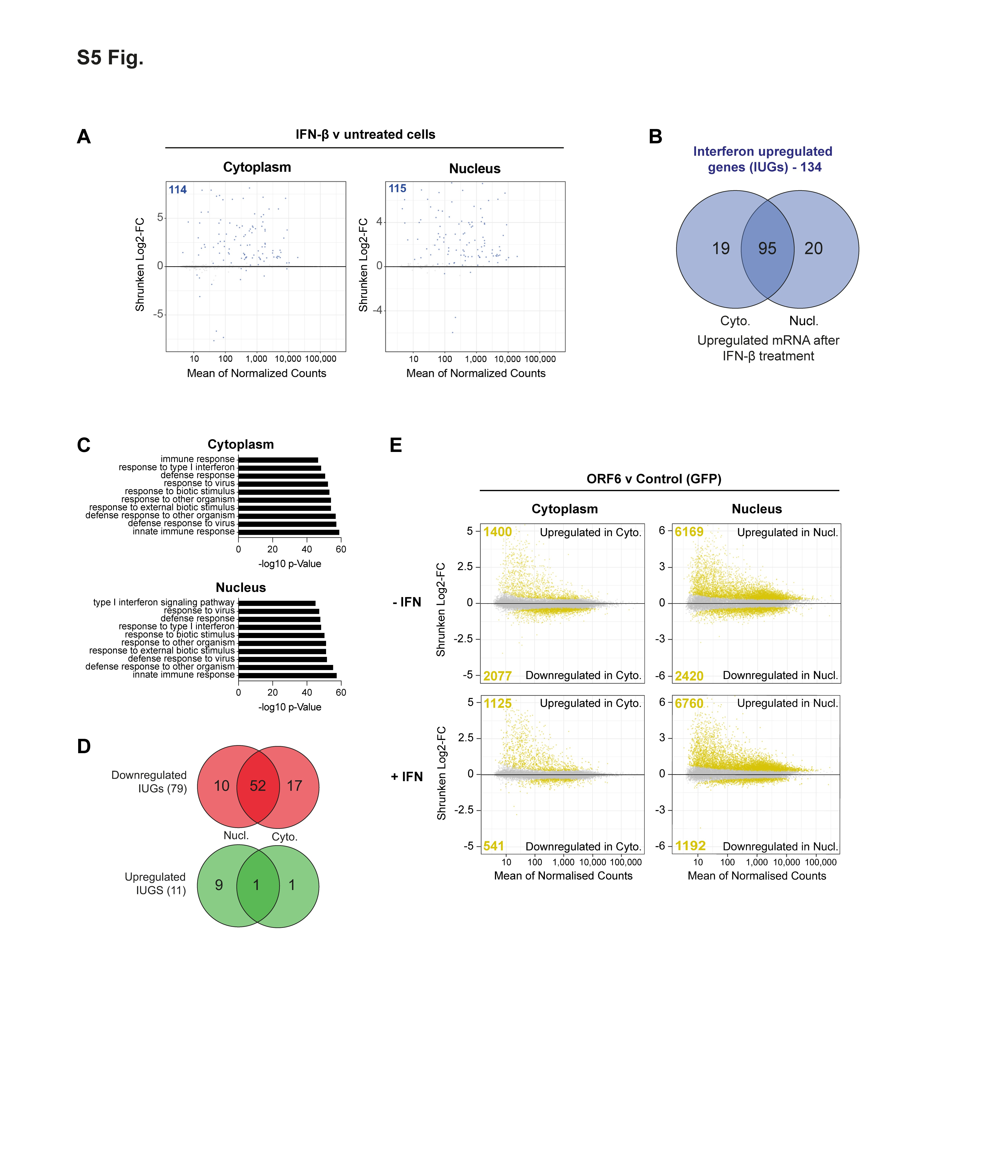

Supplement: S5 Fig — (A) From the mRNAseq data in Fig 3. The Log2-FC in mRNA abundance was compared between cells treated with IFN or left untreated in both the Cyto (left panel) and Nucl (right panel) fractions. The log-2FC was weighted against the adjusted p-value (shrunken Log2-FC) to show mRNA species that are significantly upregulated or downregulated (blue). The number of mRNAs upregulated is shown. These were designated as Interferon upregulated genes (IUGs). (B) Venn diagram showing the number of IUGs significantly upregulated in Cyto. and Nucl. fractions. (C) GO ontology enrichment analysis of significantly upregulated IUGs from A. in both the Cyto and Nucl fractions. (D) Venn diagram showing the number of IUGs significantly downregulated (red) or upregulated (green) by ORF6 in Cyto. and Nucl. fractions, as described in Fig 4A. (E) From the mRNAseq data in Fig 3. The log2-fold change (Log2-FC) in mRNA abundance was compared between ORF6 and GFP expressing cells in the Cyto. and Nucl fractions, without (upper panel) and with IFN treatment (lower panel). This log2-FC was weighted against the adjusted p-value (shrunken Log2-FC) to identify significantly upregulated/downregulated mRNAs (in yellow), with the number of mRNAs shown. The mRNA count was normalised and averaged between the three biological repeats. (TIF) [file ppat.1010349.s005.tif]

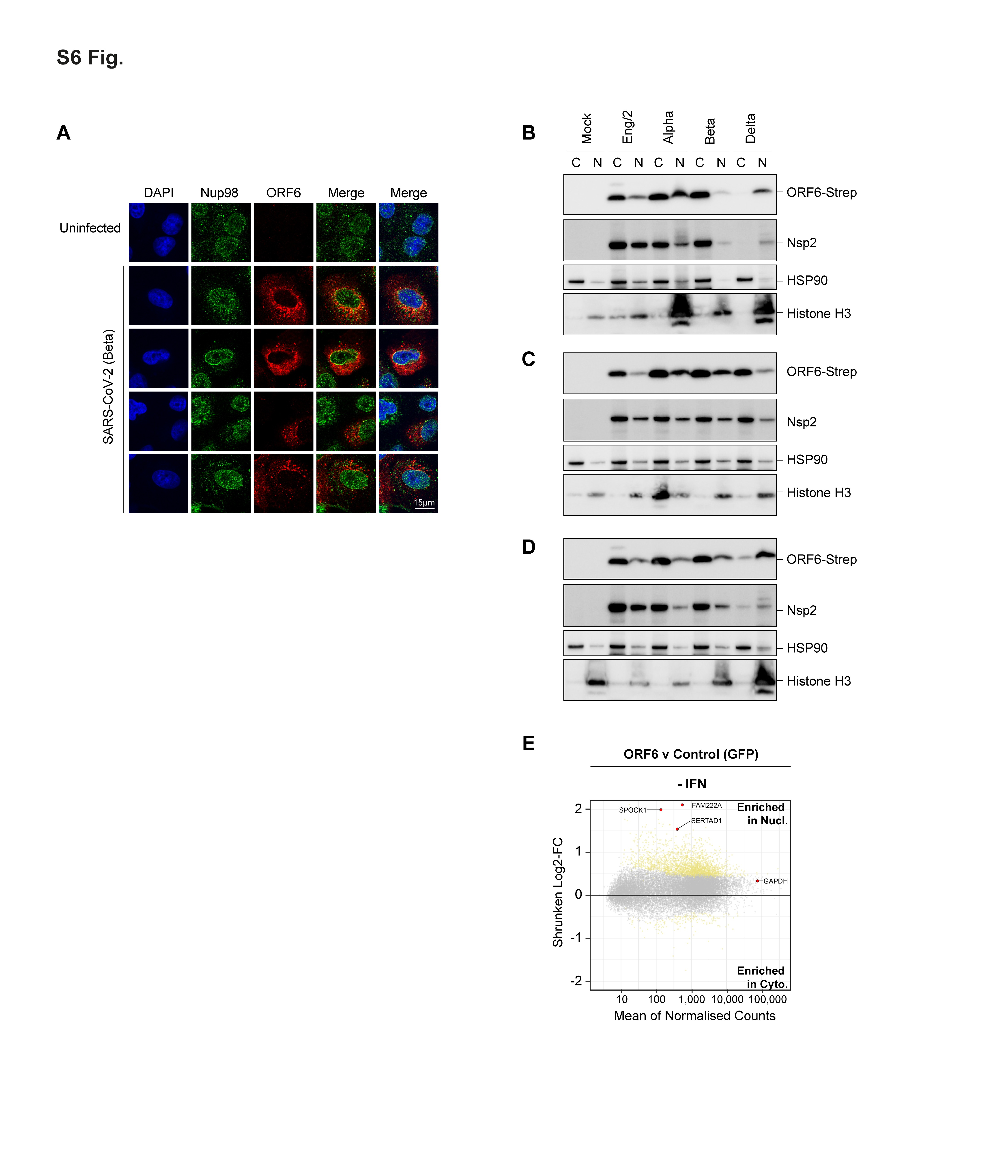

Supplement: S6 Fig — (A) Vero cells that were inoculated with SARS-CoV-2(beta) in Fig 5A were fixed, permeabilised and analysed by immunofluorescence with anti-Nup98, anti-ORF6 and DAPI. (B-D) Cell lysates from Fig 5B were analysed by immunoblotting for Strep to label ORF6, Nsp2, HSP90 (Cytoplasmic marker) and Histone H3 (Nuclear marker) to confirm successful fractionation. The analysis of three biological repeats are shown. (E) The plot shown in Fig 3B (right panel) highlighting the enrichment of the mRNA encoding the four non-antiviral proteins that were quantified by qPCR in Fig 5B. (TIF) [file ppat.1010349.s006.tif]
