# Supplementary material for: SARS-CoV-2 ORF6 disrupts innate immune signalling by inhibiting cellular mRNA export
Source: PLoS Pathog. 2022 Aug 25;18(8):e1010349. doi: 10.1371/journal.ppat.1010349 (PMC9451085; doi:10.1371/journal.ppat.1010349)
Supplement: Attachment — Submitted filename: Hall et al rebuttal letter-130522.docx [file ppat.1010349.s011.docx]

**Response to reviewer comments**

Thank you for considering our manuscript for publication. We would also like to thank all the referees for their constructive comments and we have now incorporated all of the suggested changes in the revised manuscript as detailed by point below. We believe that the extra experiments and modifications we have made have significantly improved our manuscript and hope that our amended manuscript is now acceptable for publication in PLOS Pathogens.

Thank you and best wishes,

Kate Bishop

**Reviewer 1 points:**

The previous papers by Addetia et al, mBio 2021, Miorin et al 2020 PNAS, and Kato et al, 2021 BBRC. The previous work is only briefly mentioned in the introduction and only the Addetia et al paper mentioned in the discussion as it pertains to the mRNA localization in the nucleus, not the rest of their findings. The previous work should be referenced as finding the same as the first part of this manuscript and acknowledged for the same findings.

We have now cited the above papers further in both the introduction and discussion (lines 77 and 384).

**Major:**

1. Throughout the manuscript ORF9b is used as a negative control however there is little expression of 9b in any of the western blots. This should be explained as you would want an expression control that expresses in these cells. For example, in Figure 1B, 2A, Supplementary data S1A. This should be commented on or fixed in the figures.

We have now replaced the western blot panels in Figure 1B, S1A and 2A with different exposures to show that ORF9b is indeed expressed. We have also clarified in the text where we used this expression vector compared to the pLVX-EF1α-SARS-CoV-2- ORF-2xStrep/2xStrep-ORF -IRES-Puro vector, referred to as twin-strep-tag, kindly gifted from Nevan Krogan to make it clear that the twin-strep-tag vectors express better, as seen in Fig 1D (see lines 141-145, 147 and 179-180).

2. In Figure 1F where ORF6 mutants are being expressed, there are seemingly size differences on the western blot of the mutants whilst they should have minimal changes due to the mutants being made. Is there an explanation for this difference? Are they post translationally modified that explains the weight change?

Indeed, there is a small difference in how three of the Orf6 mutants run on a gel, which is noticeable due to the small size of Orf6. All three mutants have a charged residue replaced by alanine and we expect this causes the slight shift in gel mobility. All mutants were sequenced and consistently run at these sizes.

3. In Figure 2, the western blots are much over exposed especially in Figure 2A, for example the Rae1 slice. Please provide less exposed images. Zooming in to the image shows just large black pixels leading to the idea that this is not an authentic image. The whole blot should be provided.

We thank the reviewer for pointing out the pixilation issue in the Rael panel of the western blot in Fig 2A. We presume this was an issue when saving the image and we have now replaced this panel with a lower exposure and checked that it was saved at a higher resolution.

4. In Figure 2, and throughout the whole paper, there is no reference to the fact that ORF6 is membrane associated. Where immunoprecipitations are used, does the membrane tropism of ORF6 alter the interpretation of the data? Are appropriate conditions being used to IP out of membrane fractions? Based on the model at the end of the paper, there is no realization that ORF6 is membrane associated. It is not free-floating in the cytoplasm, to the contrary as shown in the Miorin et al paper, it is ER/Golgi associated. This should be discussed, and the model changed in the paper discussion.

We can confirm that the buffer we used in the IP experiments is compatible with membrane proteins and so membrane tropism of ORF6 would not affect the data. Furthermore, a recent paper by Wong et al. has specifically studied the localisation of ORF6 and reported that it is only a peripheral membrane protein (not transmembrane) so this is unlikely to be a significant issue in the IP. Furthermore, this paper and a preprint by Yoo et al report that membrane association is not required for the nuclear transport phenotype (see comment by reviewer 2). As our model in Fig 6 is already very detailed, we feel that adding in additional membrane structures would overcrowd the image, however we have now discussed these two recent papers in the discussion (lines 396-412) to discuss the membrane association of ORF6 as requested.

5. Toward the idea of binding and localization, the Supplemental Figure 6 does show a co-localization experiment with Nup98 and ORF6. This should be quantified for association but based on this data there is little colocalization. Additional quantitation of co-localization with confocal microscopy and quantitation of overlap should be performed.

Wong et al. have recently found that ORF6 mis-localised proteins were still able to block nuclear trafficking. Indeed, much of ORF6 is found associated with membranes other than the nuclear envelope where Nup98 resides, perhaps suggesting that ORF6 has other, uncharacterised functions. We have now discussed this further in the discussion (lines 396-412).

6. The experiments on the effects of exogenous RNAs use GFP as the reporter, as in the LMB treatment experiments in Supp Figure 3. There is discontinuity in the data showing the control LMB treatment of ORF6 co-transfected cells in S3E and the western blot in S3F. In S3E, there is little GFP expression in the ORF6 expressing cells while in the western blot in S3F, there is substantial GFP bands. This discrepancy should be discussed, or more representative data shown. Is the fluorescence and western blot from the same experiment?

In this experiment, cells were imaged (Fig S3E) immediately before processing for western blots (Fig S3F) and qPCR (Fig S3G-H), so we are indeed showing different analyses of the same cells. We believe that the stronger “expression” seen in the western blots is due to the extremely high sensitivity of the anti-GFP antibody in immunoblotting and the fact that GFP folds slowly so possibly not all protein is fluorescing as soon as it can be detected in western blots. However, the order of highest and lowest GFP expression in samples is the same in both analyses. We have now included a comment on GFP expression in the text (line 232)

7. In the same experiment, the quantitation of mRNA in the nucleus and cytoplasmic fraction is quantified. The ORF6 containing cells have reduced mRNA in the cytoplasm (Supp Figure 3G) but only 3-4 fold lower than the ORF9b plasmid transfected cells. Is this enough of a difference to explain the vastly different mRNA levels described in the text and quantified in Figure S3H?

The data in Fig S3h are the same data as in Fig S3g, presented as a ratio of the absolute numbers. Therefore, yes, the differences shown in S3g are indeed enough to result in the differences in S3h.

8. In the RNASeq and fractionation section of the grant there are some analysis questions that should be further explained. The heatmap in Figure 4 has expression from +1 to -1. This is a small change for any regular RNASeq dataset analysis. Is this expected here? More discussion in the text is needed on the fold changes seen. Shown in the volcano plots in Figure 4, almost all of the genes of interest have a Log2 fold change of less than 1. This minor change should be explained in the text.

We apologise for a mislabelling of the scale for the heatmap in Figure 4 that has caused some confusion – this should have read ‘Column Z-score’ rather than ‘Expression’. The reviewer was correct in their interpretation of the data as labelled. We have now corrected the labelling. Additionally, we now provide the log2(fold change) scores represented in the figure as Supplementary Data tables. We have also expanded the description of how the genes in the heatmap were selected in the text (lines 274-277).

9. From this small difference in nuclear vs cytoplasmic difference, the fold changes seen in Figure 5 show some larger changes. The read counts of these genes should be provided in a table to show the read numbers between nucleus and cytoplasm. Are there large increases in these read numbers in total?

As noted for point 8 above, we have corrected the label in Fig 4 and now also include the log2(fold change) data as Supplementary Data tables as suggested. We apologise for the confusion caused.

10. The localization data in Figure 5 is intriguing where Delta (B.1.617.2) has a dramatically different ORF6 localization compared to the other viruses however all of the viruses tested have identical ORF6 sequences. Do you have an explanation for this difference? As stated in line 281, one idea is the viability of the cells. If taken earlier in the infection, does the staining look like the other variants tested?

We have now repeated the infections in another cell line (A549+Ace2+TMPRSS2) and included these new data as new panels in Fig 5. We see similar effects on cell viability following infection with the Delta variant as we see in Vero cells (Fig 5c), and in a subset of cells we observe altered ORF6 localisation compared to infection with the Alpha variant. As the ORF6 sequence is the same, this is likely due to another viral protein(s) but at this point we cannot say which. We have included further discussion on this point in the results (lines 340-359).

**Minor**

1. Line 68: What does “This” refer to? It seemingly references SARS-CoV-1 whilst the statement on Rae1-Nup98 goes on about SARS-CoV-2’s ORF6.

We have altered the text here for clarity (line 76).

2. Figure 1D: Add ORF9b to the list of genes that don’t express well.

As can now be seen in the updated western blot panels, ORF9b expression can be clearly detected. In case the reviewer meant ORF9c, we have now added this to our list of genes that don’t express well (line 151).

3. Line 132: It is reference 9, not reference 8 to cite here.

The citation has now been corrected

4. Line 346: provide a reference for the statement that ORF6 is in the SARS-CoV-1 virion.

The reference has now been provided.

**Reviewer 2**

**Major concerns:**

Recent studies on SARS-CoV-2 ORF6 -NUP98-RAE1 complex interaction discovered by several groups are under hot debate.
Miorin et al. proposed that ORF6 binding to the NUP98-RAE1 complex inhibits the docking of KPNB1 to the NPC. Conversely, Kato et al. suggested that aberrant nucleocytoplasmic transport is caused by the ORF6 dislocating NUP98 from the NPCs to the cytoplasm.

For instance, a research group from Stony Brook Univ. suggested decoupling SARS-CoV-2 ORF6 localization and interferon antagonism (JCS on-line). Moreover, Timothy Mitchison also confirmed the dislocation of nuceloporins which were consistent with Kato et al., which was unlikely to be directly involved in the nuclear transport inhibition by Miorin et al. (bioRxiv preprint).
What is their opinion on this key issue? They should discuss the recent JCS and Mitchison ‘s bioRxiv and deliberating their opinions on Miorin et al. & Kato et al. findings with their data.

We thank the reviewer for highlighting these interesting new papers. We have now cited the findings of these papers in our discussion and, in particular, pointed out that our data would suggest that the mechanism of inhibition proposed by Yoo and Mitchison (in which they suggest that ORF6 cross-links FG repeats in Nup98 and thus obstructs the pore in both directions) is too simplistic as we see that the ORF6 interaction with Nup98 is indirect. Rather, ORF6 binds Rael directly, suggesting inhibition via blocking RNA access to the nuclear pore (lines 396-412). From our studies, we cannot comment on Nup98 relocalisation by ORF6.

**Minor**

Not just mRNA export, Rae1-Nup98 also played key roles in cell cycle.
doi: 10.1038/nature04221. ;
doi: 10.1073/pnas.0807660105.;
doi: 10.1073/pnas.0609582104.

We have now mentioned this in the introduction and have cited the papers listed above (line 79).

**Reviewer 3**

**Major**

Since ORF6 protein inhibits the transcriptional induction of ISGs, the interpretation of mRNAseq results in IFN-treated cells is confusing. To overcome this limitation, the authors might consider to use other exogenous factors that promote transcription in a similar way in ORF6 and GFP-expressing cells.

We agree with the reviewer that the RNAseq data set is quite complex. Our original intention was to add IFN to cells in case ORF6 affected ISG mRNA export significantly more than other genes. However, as we see a strong effect on mRNA export in the absence of IFN, this control wasn’t needed for it’s original purpose and instead gave us the opportunity to look at the effect of ORF6 on ISGs both with and without IFN stimulation. We have now included text to explain this more clearly (lines 238-241). Although ORF6 can inhibit induction of ISGs, this was less pronounced in our study than the effect on mRNA export and, indeed, we saw effects on ISG mRNA export even without IFN addition. Although repeating the RNAseq in the presence of a general transcription enhancer would give us another interesting data set, RNAseq experiments are expensive and time consuming and we are not sure this would reflect the dual role of ORF6 in vivo. On this occasion we feel that the additional data gained do not justify performing this experiment as we do not believe it would affect the main conclusions of our study.

In a context of virus infection in Vero cells, several ISGs were analyzed by RT-qPCR both in the nuclear and cytoplasmic fractions. An enrichment of RIG-I and IRF1 (around 4-and 2-fold, respectively) was observed in the nucleus of SARS-CoV-2 infected vs non-infected cells, while other ISGs were not significantly enriched. Since Vero cells are deficient in IFN production, this cell line might not be the most appropriate to evaluate the inhibition of ISGs during infection and other cell lines such as Calu3 might be considered.

We agree with the reviewer that Vero cells are not ideal for studying ISG expression during infection. As suggested, we have now tested an alternative cell line that can produce IFN. We infected A549 cells that express both ACE2 and TMPRSS2 with two of our CoV-2 variants and repeated our qPCR experiments for our selected IFN-induced genes. These data are now included in Figure 5. If anything, we see a stronger effect on nuclear export in these cells with alpha infections. There was little effect in delta infections but we suspect this was because cell viability was severely affected. We have included discussion of these data in the text (lines 340-359).

**Specific points**

1. Fig. 2A. In order to draw conclusions about Gag1 protein expression, the authors may consider to normalize Gag levels by the endogenous control HSP90.

We have now normalised Gag levels to HSP90 as suggested and include the data in Figure 2A. We have also similarly normalised the Gag levels in S1 Figure and have updated the methods and figure legends accordingly.

2. Using mRNAseq, the paper shows that overexpression of ORF6 in human HEK293T cells led to 2,431 mRNA species enriched in the nucleus of ORF-6 vs GFP expressing cells. From these results, it is concluded that “ORF6 inhibits mRNA export of a broad range of cellular mRNAs”. However, the enrichment of these mRNAs in the nucleus might be caused alternatively by an increase in mRNA transcription in ORF6 vs GFP expressing cells. To exclude this possibility, cells were treated with IFN-ß, which, in principle, would promote the transcription of hundreds of IFN-stimulated genes (ISGs) and would allow to compare differences in mRNA export in ORF6 vs GFP-expressing cells. However, since ORF6 protein inhibits the transcriptional induction of ISGs, the interpretation of mRNAseq results in IFN-treated cells is confusing. To overcome this limitation, the authors might consider to use other exogenous factors that promote transcription in a similar way in ORF6 and GFP-expressing cells.

See our answer to this point in “major points” above

3. Lines 282-289. In a context of virus infection in Vero cells, several ISGs were analyzed by RT-qPCR both in the nuclear and cytoplasmic fractions. An enrichment of RIG-I and IRF1 (around 4-and 2-fold, respectively) was observed in the nucleus of SARS-CoV-2 infected vs non-infected cells, while other ISGs were not significantly enriched. Since Vero cells are deficient in IFN production, this cell line might not be the most appropriate to evaluate the inhibition of ISGs during infection and other cell lines such as Calu3 might be considered.

See our answer to this point in “major points” above

4. Line 290. From the results described in the previous point, it is concluded that “ORF6 can block the export of these mRNA species in the context of SARS-CoV-2 infection”. In addition to ORF6, SARS-CoV-2 is expressing during infection other genes with a known effect in the inhibition of mRNA nuclear export, such as nsp1 (Huang et al. J Virol. 2007). Therefore, ORF6 should not be considered the main determinant of mRNA export inhibition. To unequivocally determine whether ORF6 is responsible for this effect, a SARS-CoV-2-∆ORF6 mutant should be used.

We agree that this would be an interesting experiment and had already discussed how to perform such a study. However, we did not have a licence from the UK Government HSE to use genetically modified viruses and so were not allowed to undertake these experiments. We have now re-phrased the results (lines 335 and 363) and discussion (450) to highlight this limitation.

**Minor points**

1. Lines 59-60. Introduction. The authors may want to revise the statement with the list of SARS-CoV-2 accessory proteins according to a recent work by Jungreis et al. (SARS-CoV-2 gene content and COVID-19 mutation impact by comparing 44 Sarbecovirus genomes. Nat Commun 12, 2642. 2021). This paper found that only ORFs 3a, 3c, 6, 7a, 7b, 8 and 9b show strong protein-coding evolutionary signature, whereas ORFs 3b, 9c, and 10 lack protein-coding signatures or convincing experimental evidence of protein-coding function.

Thank you for bringing this paper to our attention. We have now included a sentence in the introduction to highlight this finding (line 61).

2. Lines 66-67. Introduction. The authors may want to revise the statement that ORF6 is the most divergent accessory gene between SARS-CoV-1 and SARS-CoV-2 ORF6. ORF6 is highly conserved in both SARS-CoV-1 and -2, while ORF 8 is one of the most divergent accessory genes.

This sentence has now been removed from the introduction.

3. Line 69. Introduction. Please note that Reference #10 describes SARS-CoV-2 Orf6 interaction with cellular Nup98, instead of SARS-CoV-1 Orf6.

Thank you for highlighting this confusion. We have amended this paragraph to clarify when we are specifically talking about SARS-CoV-1 and CoV-2 and have included the citations appropriately (line 75).

4. Line 235. mRNAs species that were significantly upregulated after IFN-β treatment were labelled “interferon upregulated genes (IUGs)”. To avoid misinterpretations, could these mRNAs be named IFN-stimulated genes (ISGs) as done by the majority of the scientific community?

We appreciate that we have used an unconventional term here. As some of the genes that were upregulated following IFN treatment in our experiment are not canonical ISGs, and some reported ISGs were not specifically identified here, we decided to refer to all the genes specifically upregulated in our study as IUGs to distinguish our gene subset from reported ISG lists. We have now written a statement to highlight and clarify this in the result section (line 274-277).
